# Toxins as biological weapons for terror—characteristics, challenges and medical countermeasures: a mini-review

**DOI:** 10.1186/s40696-016-0017-4

**Published:** 2016-04-29

**Authors:** Tamar Berger, Arik Eisenkraft, Erez Bar-Haim, Michael Kassirer, Adi Avniel Aran, Itay Fogel

**Affiliations:** 1 Surgeon General Headquarters, IDF Medical Corps, Tel Hashomer, Israel; 2 Department of Internal Medicine, Rabin Medical Center, Petah Tikva, Israel; 3 Institute for Research in Military Medicine, Faculty of Medicine, The Hebrew University, Jerusalem, Israel; 4 NBC Protection Division, IMOD, Tel-Aviv, Israel; 5 Department of Biochemistry and Molecular Genetics, Israel Institute for Biological Research, Ness Ziona, Israel; 6 Pediatric Critical Care Unit, Children’s Hospital, Montefiore Medical Center, Bronx, NY USA

**Keywords:** Toxins, Bioterror, Botulinum neurotoxin, Staphylococcal enterotoxin B, Clostridium perfringens epsilon toxin, Ricin, Medical countermeasures

## Abstract

Toxins are hazardous biochemical compounds derived from bacteria, fungi, or plants. Some have mechanisms of action and physical properties that make them amenable for use as potential warfare agents. Currently, some toxins are classified as potential biological weapons, although they have several differences from classic living bio-terror pathogens and some similarities to manmade chemical warfare agents. This review focuses on category A and B bio-terror toxins recognized by the Centers for Disease Control and Prevention: Botulinum neurotoxin, staphylococcal enterotoxin B, Clostridium perfringens epsilon toxin, and ricin. Their derivation, pathogenesis, mechanism of action, associated clinical signs and symptoms, diagnosis, and treatment are discussed in detail. Given their expected covert use, the primary diagnostic challenge in toxin exposure is the early detection of morbidity clusters, apart from background morbidity, after a relatively short incubation period. For this reason, it is important that clinicians be familiar with the clinical manifestations of toxins and the appropriate methods of management and countermeasures.

## Background

Biological warfare is defined as the intentional use of living organisms such as bacteria, viruses and fungi with the intent to cause disease, death, or environmental damage [1, 2]. Pathogens were classified by the Centers for Disease Control and Prevention (CDC) into categories A, B or C based on ease of transmission, severity of morbidity and mortality, and the likelihood of use (see also http://www.selectagents.gov/SelectAgentsandToxinsList.html and https://www.niaid.nih.gov/topics/biodefenserelated/biodefense/pages/cata.aspx). Category A bio-agents can be easily disseminated or transmitted from person to person, result in high mortality rates and have the potential for major public health impact. This might cause public panic and social disruption, and require special action for public health preparedness. Most of category A agents are considered especially dangerous due to the potential for airborne transmission. Category B bio-agents are regarded as moderately easy to disseminate, may result in moderate morbidity and low mortality, and require diagnostic capacity and enhanced disease surveillance. Category C bio-agents include emerging pathogens that could be engineered for mass dissemination because of availability, ease of production and dissemination, and have the potential for high morbidity and mortality and major health impact.

Toxins are harmful proteins derived from living organisms, mainly plants, bacteria, and fungi. Owing to their high toxicity, ease of production, and ease of dissemination, several toxins are regarded as possible bio-terror agents [3, 4]. Botulinum neurotoxin is the most potent toxin in nature and the only one included in the CDC category A [4]. Staphylococcal enterotoxin B, Clostridium perfringens epsilon toxin, and ricin are included in the CDC Category B.

The CDC categorizes natural toxins as biological agents even though there is some overlap with man-made chemical agents (toxicants). However, natural toxins are usually more toxic than chemical agents, have a longer latency period, and they are associated with a lower risk of environmental contamination [5, 6]. At the same time, there are several important differences between toxins and bio-terror pathogens: the morbidity and mortality rates are lower with toxins since they are not transmitted from person to person, and they will affect only those who were directly exposed to them following dispersion. This, together with their relatively short latency period compared to live pathogens, shortens the potential duration of the event. The non-contagious nature of toxins also reduces the need for neither healthcare surveillance nor using special protective gear by healthcare workers, apart from standard precautions. The need for environmental disinfection is minimized as the majority of toxins are sensitive to environmental conditions. Since there is no role for prophylactic treatment, there is no need in preparing large-scale points for dispensing drugs, as opposed to other live bio-terror agents [3, 6, 7].

Toxins cannot be grown in culture or identified by simple genetic sequencing of amino acids, making detection and treatment a more complex challenge. Furthermore, poisoning often presents with nonspecific clinical manifestations. Therefore, the primary diagnostic challenge in toxin exposure is to distinguish clusters of signs and symptoms from routine background morbidity. Toxins that are airborne tend to cause more severe illness than ingested toxins [8]. A high index of clinical suspicion is especially important in case of Botulinum neurotoxin poisoning; one of the few toxins for which there is an available anti-toxin and early administration is life-saving. In most cases, however, the only available treatment is supportive.

The aim of this work is to review the characteristics, diagnosis, and treatment of category A and B toxins, including Botulinum neurotoxin, staphylococcal enterotoxin B, Clostridium perfringens epsilon toxin, and ricin, in order to raise clinicians’ awareness.

## Botulinum neurotoxin

Botulinum neurotoxin (BoNT) is derived from the *Clostridium botulinum* bacterium and causes botulism. It is the most potent toxic substance currently known [9].

### Exploitation as a bio-weapon in the past

BoNT was initially used as a biowarfare agent by the Japanese during World War II. Thereafter, it was produced by other countries as well, including the USA, USSR, and Iraq. In the 1990s, the Japanese cult Aum Shinrikyo attempted to launch several terror attacks with BoNT, but all of them failed [10, 11]. Given the ease of BoNT production, there is a risk of its future use for malicious purposes.

### Toxin source

*Clostridium botulinum* is a gram-positive motile rod. There are seven serotypes of the bacterium (designated A–G) distinguished by the antigenic characteristics of the neurotoxins they produce. Serotypes A, B, E, and in rare cases, F have been described as causing disease in humans [9].

### Pathogenesis

Natural BoNT poisoning usually occurs by ingestion of formed toxin as a result of inappropriate preparation and preservation of certain food items (food-borne botulism), or sometimes by spores germinating and producing the toxin in the immature intestinal tract of infants (infant botulism). Natural poisoning can also occur as a consequence of bacterial overgrowth in necrotic wounds (wound botulism) or in individuals with a gastrointestinal disease (adult colonization botulism). Inhalational exposure to BoNT aerosol is regarded as a possible bio-terror threat. In any case, the disease develops within 12–36 h from the moment the toxin enters the body, regardless of the route of exposure [9, 12].

### Mechanism of action and resistance

BoNT is active in the neuromuscular junctions of the peripheral nervous system. It acts by blocking acetylcholine release from the presynaptic nerve endings, causing weakness and paralysis of the corresponding muscles. The interference with synaptic activity is prolonged owing to the long half-life of the toxin [10]. The toxin can be inactivated in a temperature of 85 °C for a period of 5 min, by exposure to the sun for 1–3 h, or by using chlorine solutions [13].

### Clinical manifestations

All forms of botulism lead to the same clinical syndrome of symmetrical cranial neuropathy, including diplopia, ptosis, dysphagia, xerostomia, dysphonia, or dysphasia, in combination with symmetrical descending paralysis. In most cases, there is no fever. Occasionally, gastrointestinal symptoms, such as abdominal pain, nausea, vomiting, or diarrhea, may precede the neurological presentation. The paralysis may progress to include the respiratory muscles with subsequent respiratory failure [9, 12].

### Diagnosis

The clinical diagnosis of botulism is based mainly on medical history and physical examination. In naturally-occurring botulism, the bacteria can be isolated from stool, blood, or food samples. In a bio-terror scenario, when individuals are exposed to an aerosolized toxin, traditional microbiological tests are not helpful. Gene detection methods are an option if the offending substance contains bacterial residue. The gold-standard diagnostic test is the indirect mouse neutralization bioassay (see Ref. [14] for details). The main disadvantage of this test is its 96-h duration and lack of sufficient sensitivity. A new method of endopeptidase-mass spectrometry is gaining credibility as a promising method that addresses the limitations of the mouse assay [15–19]. Electromyography can strongly support the clinical diagnosis showing a typical pattern for botulism. Findings on routine blood tests and brain imaging are usually normal, but may serve to rule out other diagnoses [9, 12, 13].

### Treatment

Treatment of botulism is mainly supportive, including prolonged mechanical ventilation. An equine-derived anti-toxin has been produced which inhibits the free toxin from binding to nerve endings, thereby preventing paralysis and respiratory insufficiency. Its efficacy diminishes once clinical signs develop, thus treatment should be administered immediately on grounds of clinical suspicion, prior to definite laboratory diagnosis. The anti-toxin is administered only in a hospital setting because of the risk of anaphylactic reaction. However, with the recent introduction of a product consisting only of the F(ab)_2_ segment of the antibody, anaphylaxis has become rare [12, 13, 20].

### Prevention

In the past, an active vaccine containing formalin-treated toxin was administered to laboratory personnel at risk of coming into contact with the toxin. The CDC has since discontinued this practice [21].

## Staphylococcal enterotoxin B

Staphylococcal enterotoxin B (SEB) is produced by the *Staphylococcus aureus* bacterium [22]. It is the only toxin of *S. aureus* that is classified as a bio-terror agent.

### Exploitation as a bio-terror agent

Inhalational exposure to SEB is extremely rare, and any event should immediately raise suspicions of malicious intent [23]. A series of laboratory accidents in the 1940s–1960s in the United States led to intensive research of the toxin which yielded a vast amount of pertinent information [24].

### Toxin source

*Staphylococcus aureus* is a gram-positive bacterium which does not produce spores and can survive in both aerobic and anaerobic conditions. It exists in nature on the skin and mucosa of humans and animals and can be found among the normal respiratory tract flora of 25–50 % of the population [25–27].

### Pathogenesis

SEB poisoning occurs mainly by consumption of contaminated food or water, and is considered a bioterror threat mainly due to the potential for an intentional use in food poisoning scenarios. Other routes of entry are the respiratory tract, the vaginal tract (via infection of a vaginal tampon, leading to the “toxic shock syndrome”), and the eyes (causing conjunctivitis). SEB is toxic in relatively minute doses [28]. It is not transmitted from person to person by contact or through air [22].

### Mechanism of action and resistance

SEB has several advantages as a bio-warfare agent, including its solubility in water, relatively high resistance to heat (it remains active even after a few minutes in boiling water) and to acidic pH levels and it is easily dispersed in air. The protein has a unique structure to sustain intestinal proteases [22, 25–27, 29]. The mechanism by which it exerts its intestinal injury is not fully clear. In the case of toxic shock syndrome it brings to a nonspecific activation of T cell lymphocytes, bypassing the specific interaction between T-cell receptors and major histocompatibility complex molecules, which results in a cytokine storm [22, 27, 30].

### Clinical manifestations

The symptoms of SEB poisoning depend on the route of exposure. The toxin is most dangerous when inhaled, causing hyperpyrexia within 3–12 h of exposure in addition to cough, rigor, headache, and myalgia. The fever may last for 2–5 days, and the cough up to 4 weeks. Inhalation of high doses may lead to acute respiratory distress syndrome (ARDS), and trigger shock and multi-organ failure [22–24, 26, 31–33]. Ingestion of SEB leads to nausea, vomiting, diarrhea, and abdominal cramps, which appear within 1–8 h of exposure, without fever or respiratory symptoms. Symptoms usually resolve within 24–48 h [22, 24, 25, 27, 31–34].

### Diagnosis

SEB can be detected by immunological assays in blood, urine, or sputum [35] up to 12–24 h from exposure; after that, it is undetectable in body fluids [24, 27]. In solid or liquid food products, SEB is detectable even in minute concentrations. Occasionally, polymerase chain reaction (PCR) is useful for detecting residual bacterial DNA in water, food, or clinical samples. Serology is usually beneficial only for retrospective detection of the toxin [36, 37].

### Treatment and prevention

Treatment is mainly supportive and does not require antibiotics. Currently, there is no available antidote or preventive vaccine, although there are several ongoing attempts at vaccine development, still in preliminary stages [34, 38–40].

## Clostridium perfringens epsilon toxin

The Clostridium perfringens Epsilon toxin (ETX) is the third most potent toxin in nature following BoNT and the tetanus toxins [31, 41]. The toxin is produced by the gram-positive, rod-shaped spore-forming anaerobic bacterium Clostridium perfringens type B and D, and impacts usually ruminants, especially young sheep, where it can cause a highly lethal enterotoxemia [41, 42].

### Exploitation as a bio-terror agent

The existing literature does not suggest that human poisoning by ETX naturally occurs even infrequently [43]. ETX is considered as a potential bioterrorism agent due to its high potency, and has been classified as a category B agent by the CDC [31, 41, 44]. Possible exposure route in case of a bioterror attack include aerosol, and food- and waterborne exposures [45].

### Toxin source

The natural habitat of the toxin-producing bacterium is the environment, and it can be found in soil, dust, sediment, litter, cadavers, and even in the digestive tract of healthy animals though in low numbers [42]. Only few ETX-mediated natural diseases have been reported in man [31, 42].

### Pathogenesis

Following rapid growth of the bacterium, such as in the intestines of young animals in which the resident digestive tract microflora is not yet developed or functional, or in cases of overeating a rich diet high in cereal crops leading to a passage of undigested fermentable carbohydrates, high production of ETX may occur, followed by enterotoxemia and injury to other target organs [42].

### Mechanism of action and resistance

ETX is produced in the exponential growth phase of the bacterium as a prototoxin. It is then converted by specific proteases to an active ETX in the intestines [41, 46]. ETX is a pore-forming toxin that increases cell permeability to small molecules and ions [31, 41]. The toxin acts locally in the intestines, increases the permeability of the gut mucosa, and then passes through and disseminates through the circulation to other organs, causing microvascular endothelial lesions mainly in the brain, the lungs and the kidneys, leading to toxic shock and death [41, 42, 46, 47]. In the kidneys it will cause interstitial hemorrhage with edema and degeneration of tubular epithelium [42, 44]. In the brain it alters the integrity of the blood–brain-barrier, causing bilateral symmetrical lesions in several brain areas consisting of perivascular edema [42, 48], resulting in foci of hemorrhage and necrosis and leading to neuronal damage [41, 42, 49]. ETX also has a direct and rapid effect in brain through targeting specific glutamatergic neurons, stimulating glutamate release and inducing neuronal cell damage [50, 51]. Once aerosolized, the toxin is stable in environmental conditions for a period of 8 h, and exposure to even a minute dose of 1 µg/kg may be fatal [45].

### Clinical manifestations

There are no available reports examining the effects of ETX on humans besides two case reports in humans published in the 1950s [31, 41]. However, it is evident that the toxin imposes risk to humans, either in natural conditions or in a bioterror setting through mass dissemination via aerosol [41, 44]. In the lack of reliable or established human data, it is assumed that there is a latent period lasting for 1–12 h between exposure and the appearance of clinical signs and symptoms. Inhalation of the toxin may result in vascular endothelial cell damage in the lungs, leading to pulmonary edema. From the lungs, the toxin will be spread to other organs, with renal, cardiovascular and central nervous system injuries, resulting in brain edema, altered consciousness, seizures, and within several hours, death will ensue [41, 44, 45].

### Diagnosis

Since introducing non-animal alternatives, the classic mouse assay involving toxin neutralization with *C. perfringens* type-specific antisera is less in use. Use of ELISA technology for specifically detecting ETX in intestinal contents is one of the best ways to confirm poisoning [52]. Quantitation of ETX protein is also possible using a novel, mass spectrometry technique [43, 53]. Mass spectrometry avoids cross-reactivity issues intrinsic in any antibody-based assay; however, ELISA and mass spectrometry do not determine whether the detected protein is biologically active. A latex agglutination test has been developed and a cytotoxicity assay using Madin–Darby canine kidney (MDCK) cells has been published [54]. PCR assays can identify the epsilon toxin gene, if it is present [43]. In the first stages of the disease, laboratory tests may show hemolytic anemia, thrombocytopenia, hypoxemia, and elevated liver enzymes. In later stages, pancytopenia is evident [45].

### Treatment and prevention

The mainstay of medical treatment is supportive care. Currently, there is a formalin-inactivated vaccine and an equine-derived antitoxin approved for animal use only. The high lethality and the rapid onset of clinical manifestations limit their efficacy [41, 55, 56]. Specific high-affinity toxin antagonists being investigated include monoclonal antibodies and selected oligonucleotides (aptamers) and dendrimer-based polymers [41, 42, 57]. Caregivers should use standard precaution measures when treating ETX casualties.

## Ricin

Ricin is produced from the *Ricinus communis* plant which is found worldwide.

### Exploitation as a bio-terror agent

Ricin was investigated as a bio-warfare agent in the 1940s in the USA and was used in the assassination of the Bulgarian journalist Georgi Markov in 1978. Ricin may also have been used in the Iran-Iraq war in the 1980s. Recently, envelopes containing ricin powder were sent by terror organizations to government personnel in Great Britain and in the USA [58–60].

### Pathogenesis

Ricin can be produced in liquid or solid forms (as a powder). It can be injected or disseminated via food, water, or air [61, 62]. All routes of exposure except ingestion are usually intentional, but human-to-human transmission does not occur [60]. Ricin is highly toxic, especially when inhaled, but it is considered less potent than other toxins [5, 59].

### Mechanism of action and resistance

Ricin penetrates the cells by binding to cell membrane receptors. It acts by inhibiting protein synthesis at the level of the ribosome [6]. Ricin is stable in environmental conditions. It can be inactivated in a temperature of 80 °C for a period of 10 min or at 50 °C for a period of 1 h, and by using chlorine solutions [5].

### Clinical manifestations

The clinical presentation of ricin poisoning depends on the dosage and route of exposure. Symptoms of inhalational exposure appear within 4–8 h and are nonspecific, including fever, cough, dyspnea, nausea, diaphoresis, and arthralgia. Studies in animals exposed to inhalational ricin reported such pathophysiological changes as necrosis and pulmonary edema leading to death from ARDS and respiratory failure within 36–72 h [63]. Respiratory symptoms do not occur in other routes of exposure, although pulmonary edema may develop due to capillary leak syndrome.

Intramuscular injection of ricin leads to local muscle necrosis and regional lymphadenopathy with minimal involvement of internal organs. Following ingestion of ricin, the clinical manifestations include: nausea and vomiting, diarrhea, hypotension, hematuria, and renal failure. Further tests reveal necrosis of intestinal epithelial cells, hemorrhage, and necrosis of the liver, spleen and kidneys. Some patients have hallucinations, seizures, and life-threatening multi-organ failure. Findings on blood and urine analysis are nonspecific: elevated levels of transaminases, lactate dehydrogenase and bilirubin, leukocytosis, metabolic acidosis, hypoglycemia or hyperglycemia, increased creatine kinase, and proteinuria. There may also be electrocardiogram changes.

Exposure to ricin via direct contact of skin or mucous membranes is not typical and may lead to erythema and pain [5, 59–61].

### Diagnosis

Clinical suspicion of inhalational exposure to ricin is based on the appearance of severe respiratory illness in a cluster of otherwise healthy individuals with a common epidemiological background. The diagnosis is made by immunoassays of clinical samples taken from nasal mucosa, skin or blood, or environmental samples taken from the point of dispersion. Serology is useful only for retrospective documentation. Occasionally, *Ricinus communis* plant DNA can be detected in products containing ricin [59, 60]. Urine can be tested for ricinine level within 2 days after exposure, since ricinine and ricin originate from the same source [62]. Studies have recently suggested the use of nanopores covered with short single-stranded DNA or RNA molecules, called aptamers, which bind to pre-selected targets with high affinity and specificity [64]. This novel method has shown high potential as both a diagnostic and a therapeutic tool [64].

### Treatment

Even before referral to the hospital, patients exposed to ricin powder should be immediately undressed and washed with soap and water. In the event of blurred vision or direct contact with the eyes, the eyes should be rinsed with water [60]. In-hospital treatment consists of rehydration, restoration to normal electrolyte levels, and respiratory support if needed. Gastric lavage or activated charcoal have not proven effective but can be used in the first hour after oral poisoning [59, 61]. Dialysis is not effective. Specific anti-toxin is currently in advanced developmental stages [59, 61].

### Prevention

Active vaccinations based on toxoid or mutant toxin are being developed. One of these is RiVax, based on a derivative of the toxin’s subunit-A chain that is enzymatically inactive and lacks the residual toxicity of the holotoxin. Animal models show it has a high efficacy and a good safety profile in humans [59, 61, 65].

## Conclusions

Toxins are a unique subgroup of bio-threat pathogens. BoNT, SEB, ETX, and ricin are considered by the CDC to be potential bioterror agents owing to their short incubation period and high toxicity. This review highlights important characteristics of these toxins in this context, as distinct from living pathogens and chemical warfare agents. These differences require different means of preparedness, diagnosis, treatment, containment, and prevention. A finding of a cluster of previously healthy individuals with severe respiratory symptoms in the absence of a known causative agent and in the appropriate epidemiological setting should raise clinical suspicions of exposure to a toxin. Clinicians need to be familiar with the unique features of toxins and their clinical manifestations in order to administer supportive care or specific treatments as soon as possible to prevent clinical deterioration and contain outbreaks.

## Abbreviations

CDC: Centers for Disease Control and Prevention
BoNT: Botulinum neurotoxin
SEB: staphylococcal enterotoxin B
ARDS: acute respiratory distress syndrome
ETX: epsilon toxin
MDCK: Madin–Darby canine kidney
